# Large central disc herniation causing cauda equina syndrome in an adolescent. A case report

**DOI:** 10.1016/j.ijscr.2020.12.066

**Published:** 2021-01-11

**Authors:** Mohamad K. Moussa, Peggy Alkefrawi, Joseph K. Elkhalil

**Affiliations:** a The Department of Orthopedic Surgery at the Lebanese University, The Faculty of Medical Sciences, Hadath, Lebanon; b The Department of Pediatrics at the Lebanese University, The Faculty of Medical Sciences, Hadath, Lebanon; c Orthopedic Surgery Department, Lebanese Geitaoui Hospital, University Medical Center, Beirut, Lebanon

**Keywords:** Cauda equina syndrome, Disc herniation, Pediatric back pain, Pediatric cauda equina

## Abstract

**Background:**

Cauda equina syndrome is a surgical emergency that requires early diagnosis and prompt intervention. It is a challenging diagnosis with a wide variety of clinical presentations that may delay the diagnosis and result in complications that severely affect the patient’s quality of life.

**Case report:**

We present a case of a 15-years-old child affected with acute cauda equina syndrome (CES) secondary to an acute disc herniation after lifting a heavy object. The patient was treated with decompressive laminectomy and discectomy that resulted in significant neurological improvement.

**Discussion:**

Etiologic factors of CES in the pediatric population include tumors, trauma, iatrogenic injuries, spinal dysraphism, constipation and inferior vena cava syndrome. This case is being reported for the rarity of this syndrome in pediatric population especially when the etiological factor is acute disc herniation, which is also rare in this particular population.

**Conclusion:**

This report is thought to increase the index of suspicion of CES in this age group especially in the presence of inciting event allowing early diagnosis and better outcomes.

## Highlights

• Cauda equina syndrome is a surgical emergency that requires early diagnosis and prompt intervention.
• Cauda equina syndrome is an extremely rare condition in the pediatric population with horrible complications if the diagnosis is delayed or missed.
• Lumbar disc herniation is relatively uncommon in children.
• In this age group, this is the first paper in the English written literature reporting a case of lumbar disc herniation complicated by CES in an adolescent.

## 1 Introduction

Although back pain in pediatrics has a wide variety of etiological factors, healthcare providers should always focus on ruling out the most dangerous situations, first. Amongst which, cauda equina syndrome (CES) represents a rare condition with horrible complications if the diagnosis is delayed or missed. The wide variety of clinical presentations of CES can delay the diagnosis [1], endangering the patient’s future quality of life and posing real medico-legal issues.

We present, herein, a case of cauda equina syndrome in an adolescent 15-years-old patient caused by acute disc herniation. At first, it showed up as a simple low back pain, then rapidly deteriorated into bilateral lower limb weakness and urinary retention.

This case was reported in line with the SCARE criteria [2].

## 2 Case presentation

This is a 15-years-old adolescent male who had initially presented to the emergency room with severe low back pain after lifting a heavy object. Physical examination of the patient was unremarkable, so he was discharged on paracetamol and muscle relaxant treatment after diagnosing the case as “benign lumbar spasm”.

**Fig. 1:**
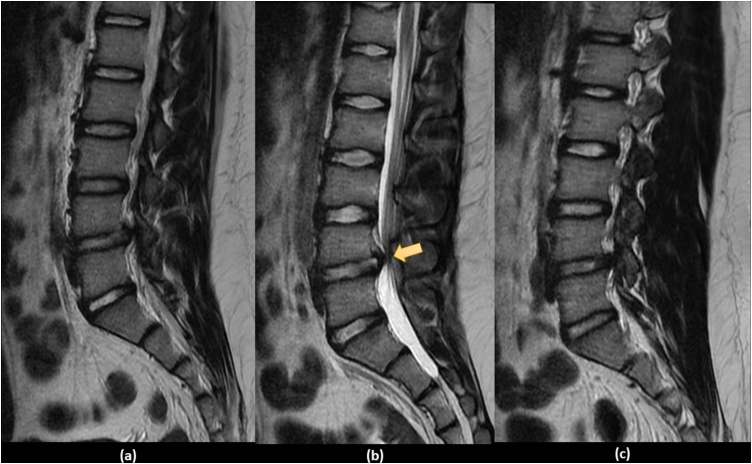
Sequential sagittal T2 weighted MRI images showing large L4 L5 disc herniation: (a) left paramedian sagittal view, (b) midsagittal view and (c) right paramedian sagittal view.

**Fig. 2:**
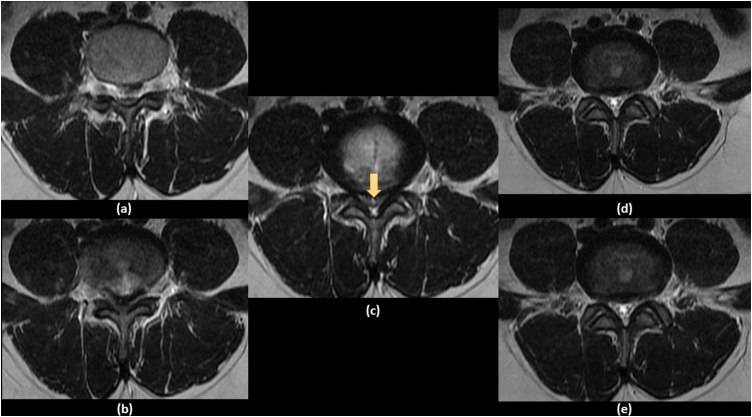
Sequential axial T2 weighted MRI images showing huge central L4 L5 disc herniation: (a) two cuts above L4 L5 disc, (b) one cut above L4 L5 disc, (c) cut at L4 L5 Disc, (d) one cut below L4 L5 disc and (d) two cuts below L4 L5 disc, (e) two cuts above L4 L5 disc.

The second day, the patient experienced a radicular type pain on both lower extremities, more severe on the right side, and then he rapidly deteriorated to have bilateral lower limb weakness and inability to get out of bed and ambulate without assistance by the 5th day of his initial consult. By the night of the 5th day, the patient became completely bed ridden. So, he was rushed to the emergency department, where physical examination showed bilateral lower limb weakness with 2/5 hip flexors, 3/5 knee extension, 2/5 ankle dorsiflexion, 1/5 long toe extensors, 2/5 ankle plantar flexors on the right side with slight better motor power on the left. The digital rectal examination revealed diminished perianal sensation and a weak anal tone. The bladder evaluation was suggestive of urinary retention. Due to the rapid progression and worsening of the clinical presentation, an urgent MRI was done showing an acute posterior central disc herniation at L4-L5 with upward migration compressing the thecal sac and L5 nerve root reducing the spinal canal to about 9 mm (Fig. 1, Fig. 2).

A diagnosis of cauda equina syndrome was made; a urinary catheter was inserted to drain 1200 cc of concentrated urine. The patient was then transferred to the operating room within 6 h of presentation; a posterior approach to the lumbar spine was utilized. After fluoroscopic localization, we exposed the lamina of L4 and L5 by subperiosteal dissection of the paraspinal muscles. The spinous process of L4 was removed totally and bilateral laminectomy L4 was done. Decompression was continued caudally so as to remove the superior portion of the lamina L5 bilaterally. The ligamentum flavum was removed, and the dural sac mobilized bilaterally to allow the identification of a large disc fragment that was removed. The L5 nerve roots were identified and freed. The wound was then copiously irrigated and closed.

Over the next few days, the patient started to have significant neurological improvement progressively (Table 1) and was discharged on the 5th day postoperatively.

**Table 1:** Physical examination finding changes of the patient pre and postoperatively.

		Pre op	Day 1	Day 2	Day 3	Day 5
Hip Flexors	***Right***	2/5	2/5	3/5	5/5	5/5
	***Left***	2/5	2/5	3/5	5/5	5/5
Knee Extensors	***Right***	3/5	4/5	4/5	5/5	5/5
	***Left***	3/5	4/5	4/5	5/5	5/5
Ankle Dorsiflexors	***Right***	2/5	2/5	2/5	4/5	4/5
	***Left***	3/5	3/5	3/5	4/5	4/5
Long toe Extensors	***Right***	1/5	1/5	1/5	2/5	3/5
	***Left***	2/5	2/5	2/5	2/5	3/5
Ankle Plantar Flexors	***Right***	2/5	3/5	3/5	3/5	4/5
	***Left***	2/5	3/5	5/5	5/5	5/5
Perianal Sensation		Disturbed			Improved	Normal
Anal Tone		Weak			Normal	Normal
Urinary function		Retention	Retention		Foley removed and passed urine	Normal
Bowel function			Passed flatus		Passed stools	Normal

## 3 Discussion

CES is a rare condition resulting from mechanical or ischemic compromise to the spinal nerve root below the conus medullaris [3]. Several studies assessed the epidemiology of this condition in the general population concluding that its incidence was best estimated in the adult age group by 7 in 100,000 [4].

However, when considering pediatric population, the CES incidence is not well known. It is merely mentioned in some few reports in record, all made during a narrow interval of time [[5], [6], [7], [8], [9], [10], [11], [12], [13], [14], [15]]. When reviewing these case reports (Table 2), tumors such as ependymoma [[5], [6], [7]] and hodgkin lymphoma [8] were most commonly cited as etiological factors followed by trauma, iatrogenic injuries, spinal dysraphism and the very rare cases of severe constipation and inferior vena cava syndrome [9,[6], [7], [8], [9], [10], [11], [12], [13], [14], [15]].

**Table 2:** Summary of cases of non-iatrogenic pediatric cauda equina syndrome reported in literature.

Summary of Cases of Non-Iatrogenic Pediatric Cauda Equina Syndrome Reported in Literature			
Authors	Age (Years)	Sex	Cause
Riffaud et al. (2003) [7]	14	Male	Epidural Hodgkin Lymphoma
Lawrentschu et al. (2005) [14]	12	Male	Constipation
Piqueras et al. (2006) [8]	10	Male	Penetrating Injury
Mohit et al. (2006) [13]	16	Female	Inferior Vena cava Thrombosis
Kabler et al. (2008) [4]	16	Male	Ependymoma of the phylum terminale
Crawford et al. (2008) [9]	6	Female	Seat Belt Injury
Estey et al. (2010) [6]	13	Male	Intradural Ependymoma
Becco de Souxza et al. (2012) [5]	13	Male	Ependymoma of the phylum terminal
**Present Case (2020)**	**15**	**Male**	**Central disc herniation**

While lumbar disc herniation is the most common cause of CES in adults [16], we were not able to find any paper reporting this condition as a causative etiology in pediatrics age group. Note that lumbar disc herniation itself is relatively uncommon in children [17]; Of 12058 Finnish child, Zitting et al. has not found any pediatric hospitalization for lumbar disc herniation below age 15, with incidence increasing to 0.1–0.2% between 15 and 20 years old [18].

Index of suspicion for CES should increase in any patient developing red flag signs such as severe low back pain, lower limbs muscle weakness, sciatica, and bowel and bladder dysfunction [16].

These signs and symptoms show up variably between patients. Whereas, in fact, urinary retention, which is one of the most specific symptoms in CES [19], can be absent in 30%–50% of cases, and often has a delayed presentation [16] (as such was the patient in our case where urinary retention manifested on the 5th day of the disease course).

This has resulted in the subdivision of CES into 3 types with a landmark symptom for each: early CES is signaled by patient presenting radiculopathy in the lower extremity, incomplete CES is marked by the addition of urinary dysfunction, and complete CES is signified with painless urinary retention and overflow incontinence. As a matter of fact, a plethora of confusing nonspecific symptoms can be added in each type making the situation vaguer and the diagnosis more challenging [3,20].

Taking in mind the severity of this medical emergency syndrome, failure to reach an accurate and timely diagnosis generates medico legal consequences [16]. In fact, delaying the treatment is associated with worse outcomes, and the best results are for patients who were treated within 48 h after developing the symptoms [21].

In consequence, high index of suspicion must be present in the emergency department in order to avoid misdiagnosing such a fearful condition, especially in the pediatric population. Even patients with “simple” back pain should be carefully examined and asked about signs and symptoms that would point out to the right and precise diagnosis.

## 4 Conclusion

So far to our knowledge, this is the first paper in the English written literature reporting a case of lumbar disc herniation complicated by CES in an adolescent. Being aware of CES in pediatric population, the time gap to making the diagnosis can be decreased allowing for prompt intervention and providing patient with the best quality of care.

## Declaration of competing interest

This article has no conflict of interest with any parties.

## Funding

This research did not receive any specific grant from funding agencies in the public, commercial, or not-for-profit sectors.

## Ethical approval

This type of study is exempt from ethical approval.

## Consent

Written informed consent was obtained from the patient for publication of this case report and accompanying images. A copy of the written consent is available for review by the Editor-in-Chief of this journal on request.

## Author’s contribution

Writing the paper: Mohamad MOUSSA, Peggy ALKEFRAWI.

Data collection: Mohamad MOUSSA.

Supervision: Joseph ELKHALIL.

## Registration of research studies

Not applicable.

## Guarantor

Dr. Joseph ELKHALIL.

## Provenance and peer review

Not commissioned, externally peer-reviewed.

## Patient perspective

The patient and his parents were satisfied with the result, and the patient returned to his regular activity 1 month post-operatively.
